# Impact of Dietary Administration of Seaweed Polysaccharide on Growth, Microbial Abundance, and Growth and Immune-Related Genes Expression of The Pacific Whiteleg Shrimp (*Litopenaeus vannamei*)

**DOI:** 10.3390/life13020344

**Published:** 2023-01-27

**Authors:** Eman M. Abbas, Ahmed Said Al-Souti, Zaki Z. Sharawy, Ehab El-Haroun, Mohamed Ashour

**Affiliations:** 1 National Institute of Oceanography and Fisheries (NIOF), Cairo 11516, Egypt; 2 Head AL Hail Aquaculture Unit, Department of Marine Science and Fisheries, College of Agriculture and Marine Science, Sultan Qaboos University, Muscat 123, Oman; 3 Fish Nutrition Research Laboratory, Animal Production Department, Faculty of Agriculture, Cairo University, Cairo 11562, Egypt

**Keywords:** *Sargassum dentifolium*, feed additives, shrimp industry, growth performances, growth-related genes, immune-related genes, stress genes

## Abstract

This work aims to determine the impact of dietary supplementation of polysaccharide, extracted from brown seaweeds *Sargassum dentifolium* on growth indices, feed utilization, biochemical compositions, microbial abundance, expressions of growth and immunity-related genes, and stress genes of the Pacific Whiteleg shrimp *Litopenaeus vannamei*. A total of 360 post-larvae of *L. vannamei* were randomly distributed into a 12-glass aquarium (40 L of each) at a stocking density of 30 shrimp with an initial weight of (0.0017 ± 0.001 g). During the 90-day experiment trial, all shrimp larvae were fed their respective diets at 10% of total body weight, three times a day. Three experimental diets were prepared with different seaweed polysaccharide (SWP) levels. The basal control diet had no polysaccharide level (SWP_0_), while SWP_1_, SWP_2_, and SWP_3_ contained polysaccharides at concentrations of 1, 2, and 3 g kg^−1^ diet, respectively. Diets supplemented with polysaccharide levels showed significant improvements in weight gain and survival rate, compared to the control diet. Whole-body biochemical composition and the microbial abundance (the total count of heterotrophic bacteria and *Vibrio* spp.) of *L. vannamei* showed significant differences among polysaccharide-treated diets compared to the control. At the end of the feeding experiment, the dietary supplementation of polysaccharide levels enhanced the expression of growth-related genes (Insulin-like growth factors (*IGF-I, IGF-II*), immune-related genes (*β* -Glucan-binding protein (*β-Bgp*), Prophenoloxidase (*ProPO*), Lysozyme (*Lys*), and *Crustin*), and stress genes (Superoxide dismutase (*SOD*) and Glutathione peroxidase (*GPx*) in the muscle tissue of *L. vannamei*. However, the current study concluded that the inclusion rate of 2 g kg^–1^ of polysaccharide as a dietary additive administration enhanced both weight gain and survival rate of *L. vannamei*, while the incorporation level of 3 g kg^–1^ reduces the abundance of pathogenic microbes and enhances the growth-, immunity- and stress-related gene expressions of *L. vannamei*.

## 1. Introduction

The shrimp aquaculture industry has experienced rapid growth and has become the most significant and leading aquaculture sector [1,2]. Although the shrimp industry has developed rapidly, the challenges faced by farmers are obtaining an increase in growth rate, low-price diets, and reducing disease outbreaks [3,4]. Furthermore, the world’s shrimp consumption has risen over the previous ten years, forcing nutrition experts to incorporate a lot of substances derived from agriculture in aquatic animal diets [1,5,6]. The Pacific Whiteleg shrimp (*Litopenaeus vannamei*) is most frequently grown worldwide, achieving more than 70% of all worldwide shrimp cultivation [7,8]. To sustain the shrimp industry worldwide, many issues must be resolved, such as poor water quality, low survivability, and diet industry improvement [9,10,11,12,13,14]. Moreover, climate change and the negative impact of environmental pollution are significant problems restricting the sustainability of aquaculture, fisheries, aquatic ecosystems, and aquatic animals so far [15,16,17,18,19,20].

Hence, shrimp diets have been expanded using several strategies to deal with such global development in the shrimp farming sector [10,21]. One of the most fundamental strategies is feed additive supplementation, which has grown to be very important for many shrimp species as a growth stimulus, immunological booster, and alternative disease resistance approach [22,23]. Aquatic plants (microalgae and seaweeds) are still widely employed in many important sectors, such as aqua-feed additives [24], plant growth enhancers [25], phytoremediation [26,27,28,29,30], human food supplement [31,32], pharmaceuticals [33,34], cosmetics substances [35,36], antimicrobial activities [37,38], and bioenergy [39,40]. As reported in 2018, the global production of seaweeds (wild captured and farmed) was about 34.4 Million Tonnes, with an industrial value of about USD 13.3 Billion [41]. This production comes from about 35 countries, while the largest producer, which produces more than 99%, is China [42]. Seaweeds have high levels of proteins, fibers, vitamins, fatty acids, minerals, pigments, and several bioactive compounds [43,44,45,46]. Among seaweed families, brown seaweeds are known as a high source of sugars, which can protect aquatic organisms from several harmful impacts while their polysaccharide has been successively used as a feed additive for Nile tilapia [47] and red tilapia [2]. The available literature has demonstrated that the polysaccharide extracted from seaweed could promote innate immunity, and enhance the resistance against pathogen infection of shrimp [48,49,50] due to its polysaccharide composition and structure (degree of branching, substituents, sulphation, and type of linkages) which are quite different from terrestrial plants [51,52] *Sargassum dentifolium*, brown seaweed is found to contain abundant polysaccharide which is a rich resource in Egypt, has been confirmed to exert multiple pharmacological properties, such as antitumor, antioxidation, hematopoiesis, immunomodulation, and gastrointestinal protection, while the dietary administration was reported to improve the non-specific immune responses in fishes [2].

Despite the importance of feed additives applied for the Pacific whiteleg shrimp, little is known about the application of polysaccharides prepared from brown seaweeds in the shrimp feed additive industry [53,54]. Immunostimulants have importance as synthetic substances that boost the immune system’s capacity to combat infections and diseases by stimulating immunological responses. Bacteria and bacterial products, complex carbohydrates, dietary factors, animal extracts, cytokines, lectins, plant extracts, and synthetic medications such as levamisole are all examples of immunostimulants that are presently available [55]. Antibiotics in the diets of cultured fish and crustaceans have been commonly used to control disease infection as well as to improve both survival and growth. However, it has been widely criticized due to the drug resistance and accumulation of chemicals in aquatic animal tissues, which can be possibly dangerous to public health. Alternatively, natural immune stimulants such as probiotics, and prebiotics are generally suggested to use in feeds to effectively promote growth and immune response, and control various diseases in aquatic animals [56].

The stimulatory effects of immunostimulants such as glucan, chitosan, nucleotides, lipopolysaccharide (LPS), sodium alginate, and other polysaccharides have been the subject of several works on fish and crustaceans [55,57].

Recently, special attention has been paid to the use of prebiotics as natural alternatives to antibiotics and immune stimulants in aquaculture. Functional polysaccharides are non-digestible ingredients because of their β-1, 3 or β-1, 4 linkages. Consumption of functional polysaccharides can reportedly improve growth performance and enhance the immune response and disease resistance of aquatic animals [58]. According to previous studies, diets containing certain polysaccharides, including medicinal plants and marine-derived polysaccharides, may improve growth rate in respect of the immune system and gastrointestinal condition in fishes and shrimps [59,60,61].

Disease resistance has been linked to an increase in cellular and humoral responses, including phagocytosis, bactericidal activity, phenoloxidase (*PO*) activity, respiratory burst, superoxide dismutase (*SOD*), and lysozyme activities in crustaceans [62]. Essential information regarding immune system activation and regulation is revealed by the expression of immune-related genes in shrimp [63]. Pattern recognition proteins (PRPs), which attach to molecules on the microbial surface, mediate the detection of invading organisms as an important step in the shrimp immune response [64].

PRP recognition of invading pathogens is a crucial intermediate step in prophenoloxidase-activating system (*ProPO*) system activation [65]. Peptidoglycan recognition proteins [66], C-type lectins [67], β-glucan-binding proteins (*β-Bgps*) [68], and lipopolysaccharide (*LPS*) and 1,3-glucan binding proteins (1,3-*Lgp*) [69] have all been described as PRPs in the *ProPO* system. The Prophenoloxidase (*ProPO*)-activating mechanism, which is triggered by PRPs binding to a microorganism’s cell wall components, is known to activate the host’s immune system [70]. Stress activates the glycolytic reactions which in turn increases the consumption of O_2_, and enhances the release of reactive oxygen species (ROS) (as hydroxyl radical, hydrogen peroxide, and superoxide anions) [71]. However, the ROS can eliminate the stressor; the increase in the ROS will cause severe destruction. Therefore, the rapid removal of excessive ROS is critical for the appropriate function of the cell. This is achieved by increasing the expression of antioxidant enzymes [72]. Superoxide dismutase (*SOD*) are antioxidant enzyme that relies on superoxide anions. Superoxide radicals are detoxified by *SOD* by being transformed into oxygen and hydrogen peroxide, which are subsequently changed into H_2_O and O_2_ by catalase and supplied to the cell as safe composites [73,74].

The copper–zinc superoxide dismutase *CuZnSOD* gene and other immune genes are also implicated in the indirect immunity of shrimp-like *Crustin*, which is essential for immunity to infections [75,76]. In *L. vannamei*, the dietary *Panax ginseng* polysaccharide extract reduces inflammation, boosts immune enzyme activity, and modifies immune gene expression [77]. A large number of genes regulate development characteristics, including growth hormone (*GH*), and insulin-like growth factors (*IGF-I* and *IGF-II*) [78]. The fast growth of *L. vannamei* aquaculture demands the creation of rapid genetic lines [79]. To the best of our knowledge, little is known about the influences of dietary polysaccharides supplementation of *Sargassum dentifolium* on shrimp growth, immunity, and stress-related gene expressions. Therefore, this study was undertaken to evaluate the effect of dietary administration of polysaccharide derived from brown seaweed (*S. dentifolium*) on growth performances, feed utilization, body composition, microbial communities, and growth, immunity, and stress genes expressions of the Whiteleg shrimp *Litopenaeus vannamei*.

## 2. Materials and Methods

### 2.1. Brown Seaweed, Sargassum dentifolium

Brown seaweed, *S. dentifolium*, was collected from Abu-Qir Bay, Alexandria, Egypt (31.3000 N and 30.1667 E) [2]. The epiphytes were removed from the obtained samples, as previously described [80]. Before use, the samples were then washed, cleaned, air-dried, powdered, and stored in plastic bags at room temperature [29]. The procedures outlined by [81] were used to extract the polysaccharide from the brown seaweed *S. dentifolium.*

### 2.2. Investigation of Water Quality

Throughout the feeding experiment, we made sure that the levels of NH_3_^−^ (mg L^−1^), NO_2_^−^ (mg L^−1^), NO_3_^−^ (mg L^−1^), alkalinity (mg L^−1^), and PO_4_^−^ (mg L^−1^) were within the ranges suggested for shrimp [82] and the guidelines of APHA [83]. In addition, daily measurements of temperature (°C), salinity (ppt), and pH were taken at 1 p.m. A thermometer hung at a depth of 30 cm was used to get an accurate reading of the water’s temperature, and a pH meter and a refractometer (Orion, Ipswich, MA, USA) were used to get accurate readings of the water’s acidity and alkalinity daily at 9.00 h.

### 2.3. The Pacific Whiteleg Shrimp (Litopenaeus vannamei)

#### 2.3.1. Animal Experiment

A private hatchery supplied post larvae (PLs) of Pacific Whiteleg shrimp *L. vannamei* to the Invertebrates Laboratory, Aquaculture Division, Suez-Branch of NIOF, Egypt. PLs were then acclimatized for 15 days in two 500-L fibreglass tanks under controlled conditions (28.0 ± 1.0 °C and salinity 29 ± 3.0 ppt) The Research Committee of the NIOF, Egypt, approved the experimental design and the adherence to ethical standards of shrimp handling.

#### 2.3.2. Experimental Design and Facilities

The current feeding trial was conducted using a completely randomized design, with triplicates. A total of 360 PLs (with an initial weight of 0.0017 ± 0.001 g) were stocked at a density of 30 shrimp in 12 glass aquariums (each with a 40 L capacity). For the 90-day feeding trial, PLs were given 10% of the total shrimp body weight three times a day (at 6:00 a.m., 12:00 p.m., and 6:00 p.m.). Each aquarium was emptied of waste and uneaten food every morning and cleaned with a siphon and 10% of the water volume was replaced with fresh, oxygenated, and filtered seawater daily [82].

#### 2.3.3. Experimental Diet

Four diets were provided to shrimp: SWP_0_: commercial shrimp diet (Aller-Aqua, Egypt, as a control basal diet, crude protein of 40% and crude lipid of 9%). The remaining three experimental diets (SWP_1_, SWP_2_, and SWP_3_) are commercial shrimp diets supplemented with 1, 2, and 3 g kg^−1^ of *S. dentifolium* polysaccharide, respectively. The additions of polysaccharide levels were performed, as previously described by Abdelrhman et al. [2]. Briefly, the commercial shrimp diet was first milled and split into three equal portions. Each polysaccharide level (1, 2, and 3 g kg^−1^) was dissolved in distilled water and then sprayed on the diet surface until complete absorption and the same adequate volume of distilled water was sprayed on the control diet (SWP_0_) without polysaccharide [84]. The sunflower oil (5 mL kg^−1^) was then sprayed over diets to cover the polysaccharide solution [85]. Finally, the diets were homogenized and re-pelletized into pellets, air-dried, placed in cellophane bags, and refrigerated at 4 °C until use.

### 2.4. Tested Parameters

#### 2.4.1. Growth Performances

At the end of the trial, the number of shrimps and weights were recorded, after 24 h of fasting, to determine the different growth indices and feed utilization using the following formulas:

(1) Weight Gain (WG, g)=FW − IW

where IW & FW are initial and final body weight (g), respectively.

(2) $$\mathrm{Specific}\;\mathrm{growth}\;\mathrm{rate}\;(\mathrm{SGR},\;\%/\mathrm{day})=100\times (\frac{\mathrm{Ln}\;\mathrm{FW}-\mathrm{Ln}\;\mathrm{IW}\;}{t})$$

where Ln and t are t natural logarithmic and time in days, respectively.

(3) $$\mathrm{Survival}\;\mathrm{Rate}\;(\%)=100\times \frac{\;\mathrm{Final}\;\mathrm{number}\;\mathrm{of}\;\mathrm{shrimp}\;}{\;\mathrm{Initial}\;\mathrm{number}\;\mathrm{of}\;\mathrm{shrimp}\;}$$

(4) $$\mathrm{Feed}\;\mathrm{conversion}\;\mathrm{ratio}\;(\mathrm{FCR})=\frac{\mathrm{Feed}\;\mathrm{intake}\;\left(g\right)\;}{\mathrm{Body}\;\mathrm{weight}\;\mathrm{gain}\;\left(g\right)}$$

#### 2.4.2. Biochemical Composition Analysis

Both experimental diets and shrimp were subjected to proximate analysis for estimating their biochemical content according to AOAC [86] guidelines prepared as detailed in the prior article [87]. To estimate the whole-body constituent (dry matter, crude fat, crude protein, and crude ash), 5 shrimp were obtained randomly from each replicate after the feeding session was completed. Shrimp were then pulverized, blended until smooth, and stored at −20 °C for further examination.

#### 2.4.3. Microbial Communities

The APHA approach [83] was used to determine the richness of microbial communities. Water (1 mL) and intestine of shrimp (1 g) samples were taken from each replicate (3 shrimp per replicate, n = 9) once the experiment was completed. Each sample (intestine and water) was inoculated with 9 mL of sterile distilled water onto plates of Trypticase soy agar (TSA) and Thio-sulphate-Citrate-Bile salts (TCBS) [88]. Plates of TSA and TCBS were incubated at 37 °C, while TCBS plates were incubated at 28 °C. Colony-forming units per milliliter were used to determine the quantity of hetero-trophic bacteria and *Vibrio* colonies present after 24 h (CFU mL^−1^) [89].

#### 2.4.4. RNA Extraction and cDNA Synthesis for Genes Expression

Triplicate samples of the shrimp’s abdominal muscles from each replicate were directly excised with fully sterile dissecting tools under cold conditions. Before performing the gene expression study, part of the muscles was frozen at −80 °C. TRIzol reagent (easy-RED, iNtRON Biotechnology) was used to extract total RNA from the shrimp’s abdomen region at the end of the experiment, as directed by the manufacturer. Using a NanoDrop system (Bio-Drop), the optical density (OD) ratio of RNA purity was determined, and 1 ng L^−1^ of RNA was used for cDNA synthesis in each reaction when the ratio was ideal (A260/A280 = 1.8). To determine the quality of the RNA, the 260/280 nm OD ratio was used. Total RNA that had been processed with DNase I (NEB, USA) was utilized as a template in a reverse transcriptase kit (RT-PCR beads, Enzynomics, Daejeon, Korea) to generate first-strand cDNA. The reaction was performed using PCR amplification (using an American product, an Applied Biosystems Veriti 96-Well Thermal Cycler) and was carried out following the manufacturer’s instructions. Real-Time PCR (Bico, Thermo-Fisher) was performed under the following cDNA conditions to detect unique and distinct products: After an initial denaturation at 95 °C for 15 min, the protein was subjected to 40 cycles at the following conditions: 95 °C for 10 s, 58–62 °C for 20 s, and 72 °C for 30 s; and finally, after the final cycle, the temperature was raised from 58–62 °C to 95 °C in increments of 0.5 °C. Primers used to probe similar genes are listed in Table 1.

The housekeeping gene (*β-actin*) was utilized to assess target gene expression or fold change [90]. When the 2^ΔΔCt^ method is used to normalize the critical threshold (Ct) quantities of the target genes with quantities of *β-actin*, the values reveal an n-fold difference in comparison to the control [91].

### 2.5. Statistical Analysis

To evaluate water quality, growth performances feed utilization indices, body composition analysis, microbial communities, and immunity and growth-related gene expression, a one-way ANOVA was employed to identify significant differences (*p* < 0.05) in the means for each variable between the polysaccharide treatments (SWP_1_, SWP_2_ and SWP_0_) and the control (SWP_0_). The statistical analysis was performed using GraphPad Prism version 9. To examine any correlation between the treatments, Tukey’s tests were utilized. Before performing the statistical analysis, all data have been checked for the normality of distribution and homogeneity of variance. Before the analysis, all data (percentages) were arc-sin transformed [92]. However, to facilitate comparisons, the data were presented as untransformed.

## 3. Results

### 3.1. Water Quality

Table 2 displays the water quality conditions recorded during feeding experiments. According to the supplied data (Table 2), the water quality was acceptable (falling under the permissible limits) for raising shrimp.

### 3.2. Growth Performances and Nutrient Utilization Indices

Table 3 demonstrates the impact of polysaccharide dietary supplementation on shrimp growth, survival, and feed utilization. Compared to SWP_0_, Table 3 showed that SWP_1_, SWP_2_, and SWP_3_ demonstrated significant (*p* < 0.05) increases in WG. Moreover, SWP_1_ and SWP_2_ showed significant (*p* < 0.05) increases in SR, while SWP_3_ showed significant (*p* < 0.05) decreases, compared to SWP_0_. On the other hand, there were no significant differences (*p* < 0.05) in SGR or FCR across the supplemented diets (SWP_0_, SWP_1_, SWP_2_, and SWP_3_), as presented in Table 3.

### 3.3. Shrimp Body Composition Analysis

The body composition analysis of the content (% of dry weight) of protein, fat, ash, and dry matter is presented in Table 4. The highest significant (*p* < 0.05) values of protein and dry matter were reported by SWP_0_ followed by SWP_1_, SWP_3_, and SWP_2_, while the highest significant (*p* < 0.05) values of fat and ash were reported by SWP_2_ followed by SWP_3_, SWP_1_, and SWP_0_ (Table 4).

### 3.4. Microbial Communities

Table 5 shows the impact of experimental diets supplemented with different concentrations of the polysaccharide (SWP_1_, SWP_2_, and SWP_3_), compared to SWP_0_, on the total count of THB and TVC in both the water and intestine of shrimp. The data showed that the abundance of microbes (THB and TVC) was higher in the intestine than in water. Compared to SWP_0_ both THB and TVC count in both water and intestine gradually decreased as polysaccharide levels increased (Table 5).

### 3.5. Growth, Immunity, and Stress-Related Genes Expressions

**Figure 1:**
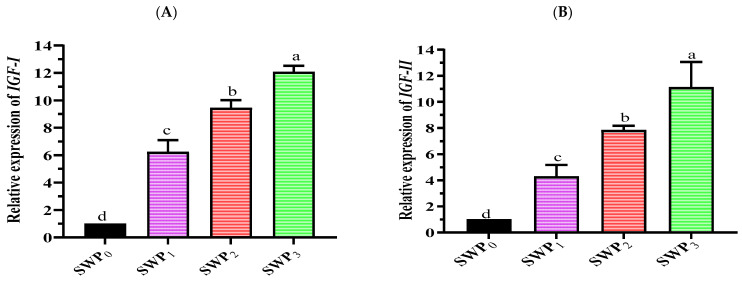

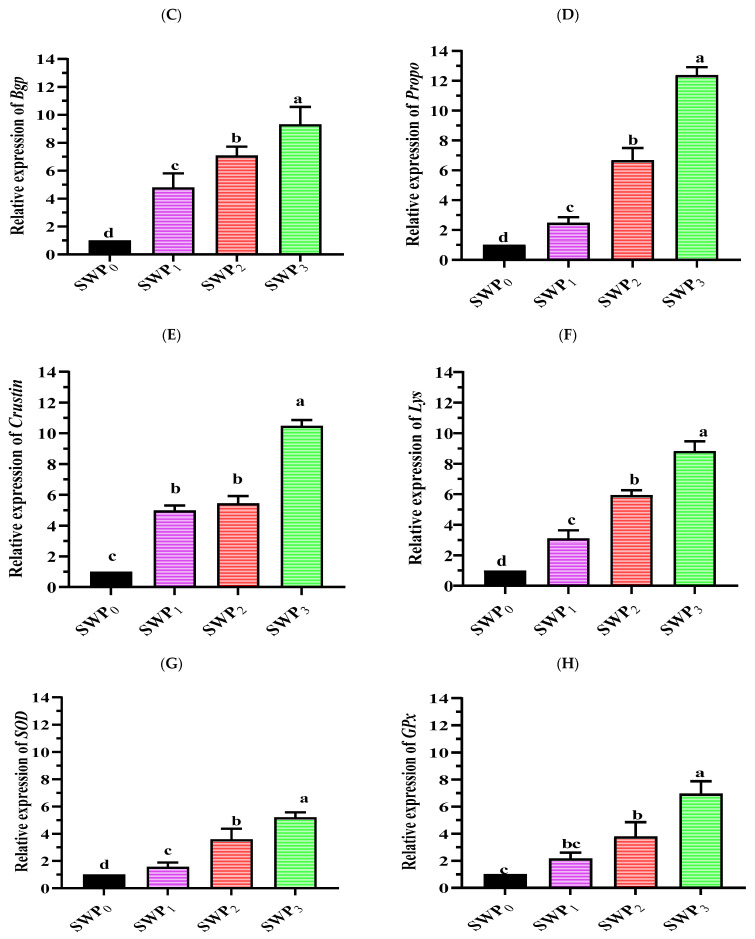
Analysis of gene expressions of growth-related genes [*IGF-I* (**A**) and *IGF-II* (**B**)], immune-related genes [*Bgp* (**C**), *ProPO* (**D**), *Crustin* (**E**), and *Lys* (**F**)], and stress genes [(*SOD* (**G**) and *GPx* (**H**)], compared to the expression of a housekeeping gene (β-actin gene) in the different dietary supplementation of polysaccharide-extracted from brown seaweed, *S. dentifolium*. The presented data are Means ± SD (*n* = 3). Different letters are significantly different (*p* < 0.05). In general, the commercial diet supplemented with 3 g kg^−1^ of polysaccharide produced from brown seaweed *S. dentifolium* resulted in the highest expression of the eight genes (*p* < 0.05) compared to the other diets examined.

At the end of the experiment, the dietary supplementation of polysaccharides enhanced the expressions of immune-related, growth-related, and stress genes in the muscle tissue of *L. vannamei*. Regarding the expressions of growth-related genes (*IGF-I* and *IGF-II*), their expressions were considerably up-regulated (*p* < 0.05) in the treatments with the different polysaccharide concentrations compared to the control (SWP_0_). The expression was increased in the SWP_3_ and found to be higher than the SWP_0_ with approximately 12 and 11-fold change, respectively (Figure 1A,B). The expressions of immune-related genes (*Bgp, ProPO, Crustin,* and *Lys*) were markedly up-regulated in the SWP_3_ treatment where the fold changes were 9.3, 12.4, 10.5, and 8.8, respectively, which were higher than SWP_0_ (Figure 1C–F).

Compared to the control group (SWP_0_), the *ProPO* gene exhibits the highest expression levels across all treatment concentrations. For the *Crustin* gene, there is a significant difference between the SWP_3_ treatment and the control, while there was no significant difference between the SWP_1_ and SWP_2_ and the control. Furthermore, the expression of stress genes (*SOD* and *GPx*) in SWP_3_ were considerably increased by 5.2- and 6.9-folds, respectively, relative to the control (Figure 1G,H). However, there was a significant difference (*p* < 0.05) in *SOD* gene expression among all treatments compared to the control with more increase in the SWP_3_ treatment. Meanwhile, there was a significant difference (*p* < 0.05) in the gene expression of *GPx* between SWP_3_ and SWP_2_ treatments relative to the control SWP_0_, but no significant difference was observed between SWP_2_ and SWP_1_ treatments.

## 4. Discussion

Seaweed polysaccharides are recognized as high-value active molecules that improve growth performances, enhance the immune system response, and have many health benefits for aquaculture organisms [2,54,93,94,95,96,97]. In the present study, we hypothesized that the dietary administration of polysaccharide derived from brown seaweed (*Sargassum dentifolium*) ameliorates the growth performances, feed utilization, body composition, microbial communities, and growth, immunity, and stress genes expressions of the Whiteleg shrimp *L. vannamei*. The current feeding trial demonstrated that the weight gain of *L. vannamei* was improved significantly with increasing polysaccharide levels in the commercial diet compared to the control diet. The present findings are parallel to the previous studies conducted on different shrimp and fish species. For example, Lee et al. [98] reported that the hot-water extract of the brown seaweed *Sargassum horneri* significantly improves growth performances, stimulates innate immunities, and enhances immune gene expressions of shrimp *L. vannamei* and recommended that the ideal inclusion level is 5 g kg^–1^. Additionally, the study by Liu et al. [99] investigated the impact of different inclusion levels (0, 1, 2, and 3 g kg^–1^) of polysaccharides extracted from green seaweed (*Enteromorpha*) into the diet of banana shrimp *F. merguiensis* and concluded that 1 g kg^–1^ significantly enhances growth performance, improves nonspecific immunity, and modulates the intestinal function of *F. merguiensis*, while Abdelrhman et al. [2] investigated the effect of different dietary inclusion rates (0, 10, 20, and 30 g kg^–1^) of polysaccharides obtained from brown seaweed *S. dentifolium* on the hybrid red tilapia, and concluded that the 30 g kg^–1^ level achieved the highest significant growth performance, FCR, and hematological indices. However, the inconsistency in the inclusion levels among these studies may be due to the different initial weight, seaweed species, species (fish and shrimp species), age, etc.

Gut microbiota abundance rapidly responds to variations in dietary intake, composition, and components. Therefore, it has a huge impact on the health benefits of all aquatic organisms such as food consumption, digestion, nutrient utilization, absorption, and immunity responses [22,87,100,101]. At present, the evaluation of disease resistance is important in the aquaculture industry, the blood antioxidant and immune factor activity is a good health status indicator for investigating the immune response and disease resistance in *L. vannamei* such as white spot syndrome virus (WSSV) [49] and *Vibrio alginolyticus* [102]. Reactive oxygen species (ROS) are chemically reactive molecules containing oxygen, such as oxygen ions and peroxides. Excessive amounts of ROS can affect the structure and stability of functional proteins, unsaturated fatty acids, and nucleic acids, causing oxidative damage to the immune system of the organism and increasing the susceptibility to pathogens in shrimp [73]. Hence, the health of aquatic organisms depends on the balance between the production of ROS and antioxidant enzymes such as SOD and GPx which protect the animal cells against free radicals. The current findings showed that dietary polysaccharides derived from brown seaweed (*S. dentifolium*) effectively improved the activities of antioxidant enzymes, including *SOD* and *GPx*. Similarly, the *SOD* and *GPx* activities of different crustaceans were increased after feeding diets supplemented with *Angelica sinensis* polysaccharides in whiteleg shrimp [60] and *β-glucan* [103], and *Rhodiola rosea* polysaccharides in red swamp crayfish [104].

The current work reported that, compared to SWP_0_, the THB and TVC counts were significantly (*p* > 0.05) decreased with the increase in the inclusion levels of polysaccharides (SWP_1_, SWP_2_, and SWP_3_). These results are in agreement with those reported in the study by Mansour et al. [87] who found that the increasing levels of astaxanthin, extracted from the cyanobacterium strain, *Arthrospira platensis* NIOF17/003, in *L. vannamei* diet significantly (*p* > 0.05) decreased the counts of THB and TVC. However, the action mechanism of how seaweed-polysaccharide affected the abundance of microbiota is still not clear and requires further studies [87,101].

Several genes involved in immunological response were the focus of the current investigation. In SWP_3_ treatment, the up-regulatory gene expression was noticeably higher. Results showed increased expression with the treatments compared to the control (SWP_0_), suggesting that the polysaccharide can improve the immune status of shrimp through microbial cell walls composed of peptidoglycans, lipopolysaccharides (LPS), and β-1, 3-glucans, which can activate the shrimp immune response by triggering the main non-specific defense mechanism [22,87,105,106].

Prophenoloxidase is a crucial enzyme in invertebrate humoral immunity that promotes melanization to get rid of invasive pathogens [107], and is linked to cuticle sclerotization and wound healing [108]. Invertebrates have a non-self-recognition system called the ProPO activation system, which may detect and react to intruders using peptidoglycan or lipopolysaccharides from bacteria and β-1, 3-glucans from fungi [109]. The mRNA expression of the *ProPO* gene was shown to be considerably higher across all treatments compared to the control group, and this expression was found to be the greatest among all the investigated genes as seaweed polysaccharide content was increased (3 g kg^–1^ diet). Feeding *P. monodon* shrimp a diet that included the polysaccharide fucoidan from the brown seaweed *S. wightii* increased the expression of the *ProPO* gene [110]. Some other dietary supplements derived from microalgae and seaweeds raised the shrimp’s *ProPO* system and improved the humoral immune response. Our findings are consistent with prior studies conducted on *L. vannamei* [22,87].

*Crustin*, defined as part of the innate immune system [111], is a protein found in the hemocyte granules of crustaceans and is effective against several microorganisms. In this study, supplemented diets of the extracted polysaccharide increased *Crustin* gene expression, and there was a clear difference between the three treatments. Significant elevation of *Crustin* mRNA levels in *Marsupenaeus japonicus* has been observed after the administration of peptidoglycan [112]. The *Crustin* gene was upregulated (*p* < 0.05) in Pacific white shrimp *L. vannamei* administered supplemental astaxanthin [87,113]. As a protein found in eukaryotes and prokaryotes, lysozyme has been around for quite some time and is considered to be one of the earliest known antibacterial proteins [114]. Non-specific innate immunity relies on its ability to break down the b -1,4 glycosidic link between N-acetylmuramic acid and N-acetylglucosamine in bacterial cell wall peptdoglycan [115].

In the current investigation, *Lys* gene expression was shown to be considerably greater in the treatment groups (SWP_1_, SWP_2_, and SWP_3_) than in the control group (SWP_0_). Another transcriptome investigation using species that face environmental challenges also produced similar findings [116,117]. These findings demonstrated that lysozyme is a crucial part of the shrimp’s anti-bacterial defense mechanism and is evoked by a variety of immunostimulating substances. The antioxidant enzymes catalase and glutathione peroxidase convert hydrogen peroxide into oxygen and water, while *SOD*, one of the stress genes, is involved in the elimination of superoxide anions by converting them into hydrogen peroxide and water [118]. Consequently, these antioxidant enzymes give post-phagocytosis self-protection to the hemocytes of oxygen-respiring animals, hence preserving the organisms’ health and viability [119,120]. Compared to the control, the expression of the *SOD* gene was elevated in the three experimental conditions, and previous research [22,87,113,116] indicated that the feeding additive increased the expression of the *SOD* gene, which is involved in the antioxidant enzyme system in *L. vannamei*.

In the glutathione defense system, *GPx* is responsible for the reduction of hydrogen peroxide to water [117,121]. In our investigation, the expression of *GPx* was found to be higher in the SWP_3_ treatment where a higher concentration of seaweed polysaccharides was used. Thus, both stress genes in this study are significantly upregulated in comparison to the control group, and the activities of the *SOD* and *GPx* increase together with an increase in superoxide anion (O_2_^−^) and hydrogen peroxide (H_2_O_2_), which may indicate increases in the activity of NADPH-oxidase and the production of a mass of reactive oxygen species (ROS) that can represent as a defense mechanism against microbial infection [73,122]. Recent research has evaluated the expression of genes involved in immunity in shrimp [123,124] and has concentrated on ways to boost their natural defenses.

There are two types of insulin-like growth factor (*IGF*) peptide hormones, *IGF-I* and *IGF-II*; there are also cell surface receptors and circulating binding proteins. *IGF-II*, like *IGF-I*, has a role in protein metabolism, cellular differentiation, cell proliferation, and somatic growth. Based on the findings of the current study, it appears that seaweed polysaccharide extraction may increase the expression of growth-related genes at the mRNA level, hence boosting growth capacity indirectly. Other studies examining the impact of employing different carbon sources for boosting *IGF-I* and *IGF-II* gene expression revealed similar outcomes [123]. Furthermore, utilizing the green microalga, *T. suecica,* and *A. platensis* nanoparticles as the supplementary feeds for *L. vannamei* greatly increased the expression of both genes and improved growth [22,100].

## 5. Conclusions

Globally, shrimp diets have expanded by using several strategies to deal with the development in the farming of the Pacific whiteleg shrimp *L. vannamei*. Despite the importance of feed additives for *L. vannamei*, little is known about the application of polysaccharides prepared from brown seaweeds in the *L. vannamei* feed additive industry. In the current work, the inclusion rate of 2 g kg^–1^ of polysaccharides, a high-value active molecule prepared from brown seaweed *Sargassum dentifolium*, as dietary additive administration enhances final weight gain and survival rate of the Pacific Whiteleg shrimp, *L. vannamei*, while incorporation level of 3 g kg^–1^ reduces the abundance of pathogenic microbes, moreover, enhances the immunity and stress-related gene expressions of *L. vannamei*. However, further studies should be conducted to maximize the benefits of polysaccharides prepared from seaweed species as additive administrations to the Pacific whiteleg shrimp *L. vannamei*.

## Figures and Tables

**Table 1:** Oligonucleotide primer sequences applied in RT-PCR for immune-related, antioxidant genes and growth-related genes.

Genes	Sequences	Amplicon Size
*β-actin* (AF300705)	F: GCCCATCTACGAGGGATA R: GGTGGTCGTGAAGGTGTAA	121 bp
*Bgp* (AY249858)	F: ACGAGAACGGACAAGAAGTG R: TTCAGCATAGAAGCCATCAGG	137 bp
*ProPO* (AY723296)	F: CGGTGACAAAGTTCCTCTTC R: GCAGGTCGCCGTAGTAAG	122 bp
*Crustin* (AF430076)	F: ACGAGGCAACCATGAAGG R: AACCACCACCAACACCTAC	141
*Lys* (AY170126)	F: GGACTACGGCATCTTCCAGA R: ATCGGACATCAGATCGGAAC	97 bp
*IGF-I* (KP420228) *	F: GTGGGCAGGGACCAAATC R: TCAGTTACCACCAGCGATT	123 bp
*IGF-II* (XM02739466) *	F: CTCTGTACAGTCAGCCCAGC R: CACACCCAGTCAGTCCCAAG	220 bp
*SOD* (DQ005531)	F: AATTGGAGTGAAAGGCTCTGGCT R: ACGGAGGTTCTTGTACTGAAGGT	153
*GPx* (AY973252)	F: AGG GACTTC CAC CAG ATG R: CAA CAACTC CCC TTC GGTA	117

* Designed by NCBI tool.

**Table 2:** Water quality parameters of experimental diets.

Tested Parameters	SWP_0_	SWP_1_	SWP_2_	SWP_3_
NH_3_^−^ (mg L^−1^)	0.119 ± 0.001	0.106 ± 0.016	0.123 ± 0.015	0.125 ± 0.004
NO_2_^−^ (mg L^−1^)	0.119 ± 0.016 ^bc^	0.101 ± 0.009 ^c^	0.140 ± 0.001 ^a^	0.132 ± 0.003 ^ab^
NO_3_^−^ (mg L^−1^)	0.222 ± 0.028	0.217 ± 0.008	0.262 ± 0.004	0.257 ± 0.005
PO_4_^−^ (mg L^−1^)	0.485 ± 0.010	0.495 ± 0.018	0.505 ± 0.007	0.485 ± 0.018
Alkalinity (mg L^−1^)	7.700 ± 0.625 ^b^	7.625 ± 0.050 ^b^	8.563 ± 0.438 ^ab^	9.038 ± 0.763 ^a^
Temperature (°C)	26.84 ± 0.20 ^a^	26.75 ± 0.07 ^a^	26.46 ± 0.04 ^b^	26.65 ± 0.15 ^ab^
Salinity (ppt)	32.25 ± 0.09 ^b^	32.35 ± 0.03 ^b^	32.46 ± 0.02 ^a^	32.32 ± 0.04 ^b^
pH	7.79 ± 0.02 ^a^	7.82 ± 0.01 ^a^	7.78 ± 0.03 ^a^	7.73 ± 0.08 ^b^

SWP_0_, SWP_1_, SWP_2_, and SWP_3_: diets supplemented with 0, 1, 2, and 3 g of polysaccharide extracted from brown seaweed *S. dentifolium*. The presented data are Means ± SD (*n* = 3). Different letters in the same column are significantly different (*p* < 0.05). The absence of letters in the same row means that there are no significant differences.

**Table 3:** Growth performance and feed utilization of shrimp *L. vannamei* fed on experimental diets.

Indicator	SWP_0_	SWP_1_	SWP_2_	SWP_3_
IW (g)	0.0017 ± 0.001	0.0017 ± 0.001	0.0017 ± 0.001	0.0017 ± 0.001
WG (g)	10.43 ± 1.15 ^c^	12.75 ± 2.21 ^b^	14.97 ± 1.26 ^a^	15.06 ± 1.28 ^a^
SR (%)	75.56 ± 2.94 ^b^	77.78 ± 4.08 ^a^	83.33 ± 3.74 ^a^	60.00 ± 2.45 ^c^
SGR	7.29 ± 0.55	7.45 ± 0.77	7.59 ± 0.33	7.59 ± 0.41
FCR	1.58 ± 0.05	1.59 ± 0.07	1.59 ± 0.09	1.58 ± 0.15

SWP_0_, SWP_1_, SWP_2_, and SWP_3_: diets supplemented with 0, 1, 2, and 3 g of polysaccharide extracted from brown seaweed *S. dentifolium*. The presented data are Means ± SD (*n* = 3). Different letters in the same column are significantly different (*p* < 0.05). The absence of letters in the same row means that there are no significant differences.

**Table 4:** Composition analysis (%) of shrimp *L. vannamei* fed on different experimental diets.

Diets	Composition Analysis (% of Dry Weight)			
	Dry Matter	Protein	Fat	Ash
SWP_0_	26.53 ± 0.13 ^a^	23.12 ± 0.03 ^a^	7.79 ± 0.01 ^d^	1.60 ± 0.01 ^d^
SWP_1_	25.33 ± 0.04 ^b^	22.32 ± 0.03 ^b^	10.61 ± 0.02 ^c^	1.89 ± 0.01 ^c^
SWP_2_	24.60 ± 0.03 ^d^	21.88 ± 0.02 ^d^	11.00 ± 0.01 ^a^	2.48 ± 0.02 ^a^
SWP_3_	24.93 ± 0.04 ^c^	22.10 ± 0.01 ^c^	10.78 ± 0.03 ^b^	2.19 ± 0.01 ^b^

SWP_0_, SWP_1_, SWP_2_, and SWP_3_: diets supplemented with 0, 1, 2, and 3 g of polysaccharide extracted from brown seaweed *S. dentifolium*. The presented data are Means ± SD (*n* = 3). Different letters in the same column are significantly different (*p* < 0.05). The absence of letters in the same row means that there are no significant differences.

**Table 5:** Effect of brown seaweed polysaccharide on the bacterial abundance in water and intestine of *L. vannamei*, total heterotrophic bacteria (THB), total vibrio count (TVC), and TVC/THB ratio.

Bacterial Count (CFU mL^−1^ × 10^5^)	Experimental Diets			
	SWP_0_	SWP_1_	SWP_2_	SWP_3_
Water				
THB	7.251 ± 0.0033 ^d^	4.200 ± 0.0030 ^c^	2.651 ± 0.0063 ^b^	0.119 ± 0.0066 ^a^
TVC	0.114 ± 0.0005 ^d^	0.068 ± 0.0002 ^c^	0.045 ± 0.0003 ^b^	0.005 ± 0.0004 ^a^
Intestine				
THB	80.00 ± 0.0033 ^d^	50.00 ± 0.0035 ^c^	35.00 ± 0.0020 ^b^	3.00 ± 0.0033 ^a^
TVC	0.591 ± 0.4583 ^d^	0.476 ± 0.4041 ^c^	0.282 ± 0.5508 ^b^	0.007 ± 0.0306 ^a^

SWP_0_, SWP_1_, SWP_2_, and SWP_3_: diets supplemented with 0, 1, 2, and 3 g of polysaccharide extracted from brown seaweed *S. dentifolium*. The presented data are Means ± SD (*n* = 3). Different letters in the same column are significantly different (*p* < 0.05). The absence of letters in the same row means that there are no significant differences.

## Data Availability

Not applicable.
